# Multi-ingredient pre-workout supplementation changes energy system contribution and improves performance during high-intensity intermittent exercise in physically active individuals: a double-blind and placebo controlled study

**DOI:** 10.1186/s12970-020-00357-6

**Published:** 2020-06-03

**Authors:** Caique Figueiredo, Fábio Santos Lira, Fabricio Eduardo Rossi, François Billaut, Rodrigo Loschi, Camila S. Padilha

**Affiliations:** 1 Exercise and Immunometabolism Research Group, Department of Physical Education, School of Technology and Sciences, Presidente Prudente, São Paulo, Brazil; Post Graduation Program in Physical Therapy, São Paulo State University (UNESP), São Paulo, Brazil; 2 Immunometabolism of Skeletal Muscle and Exercise Research Group, Department of Physical Education and Associate Graduate Program in Health Science, Federal University of Piauí (UFPI), Teresina, PI Brazil; 3 Department of Kinesiology, Laval University, Laval, Quebec, QC Canada; 4 VP Centro de Nutrição funcional, São Paulo, Brazil

**Keywords:** Pre-workout drink, Performance fitness, Energy expenditure, Ergogenic aid

## Abstract

**Background:**

Nutritional ergogenic aids are commonly used to boost physiological adaptations to exercise and promote greater fitness gains. However, there is a paucity of data about multi-ingredient pre-workout supplementation (MIPS). Therefore, the aim of the present study was to investigate the acute effects of MIPS on the oxidative, glycolytic and ATP-CP energy systems contribution, time spent above 90% V̇O_2max_ (T90% V̇O_2max_), excess post-exercise oxygen consumption (EPOC) magnitude, number of efforts and time to exhaustion during a high-intensity interval exercise (HIIE) session.

**Methods:**

Twelve physically-active and healthy men completed the HIIE sessions that involved running bouts of 15 s on the treadmill at 120% of the maximum aerobic speed (MAS), interspersed with 15 s of passive recovery. Blood lactate was collected at immediately post, 3, 5, and 7 min post exercise. The contribution of ATP-CP, glycolytic and oxidative systems was analyzed at rest, during the HIIE sessions and for 20 min post. Performance variables (time to exhaustion, number of efforts) and oxygen consumption were also analyzed.

**Results:**

MIPS significantly increased the number of efforts performed (MIPS: 41 ± 10 vs Placebo: 36 ± 12, *p* = 0.0220) and time to exhaustion (MIPS: 20.1 ± 6 min vs Placebo: 17 ± 5 min, *p* = 0.0226). There was no difference between supplements for both T90% V̇O_2max_ (*p* = 0.9705) and EPOC (*p* = 0.4930). Consuming MIPS significantly increased the absolute oxidative energy system contribution by 23.8% (*p* = 0.0163) and the absolute ATP-CP contribution by 28.4% (*p* = 0.0055) compared to placebo. There was only a non-significant tendency for a higher glycolytic system contribution after MIPS ingestion (*p* = 0.0683).

**Conclusion:**

Acute MIPS ingestion appears effective at increasing both aerobic and anaerobic alactic energy contribution and time to exhaustion during a HIIE protocol.

## Background

High-intensity intermittent exercise (HIIE) is an excellent strategy for increasing maximal oxygen uptake (V̇O_2max_) and decreasing body fat [1, 2]. HIIE protocols involving relatively short efforts (< 1 min) performed at an intensity corresponding to or above 100% of maximal aerobic speed (MAS) have been frequently used to increase aerobic power [3, 4]. The effectiveness of HIIE is attributed to, at least in part, a higher energy expenditure and number of efforts near V̇O_2max_ (90–100%)_._ Indeed, these factors are important to increase cardiorespiratory fitness [3, 5]. Higher cardiorespiratory fitness is related to higher physical performance and reduced risk of cardiovascular diseases [6, 7]. Therefore, strategies that optimize energy expenditure during a HIIE session are extremely important for reducing excess body fat accumulation [8].

Nutritional ergogenic aids are commonly used to boost these physiological adaptations to exercise and to promote greater fitness gains. In this perspective, some exercise modalities have been combined with multi-ingredient pre-workout supplementation (MIPS; typically containing creatine, arginine, leucine, valine, isoleucine, tyrosine and caffeine) to enhance sympathetic response, substrate availability, and muscle contraction [9–11]. Acute ingestion of MIPS has been demonstrated to optimize performance during continuous runs to exhaustion [11] and during tests of explosive power, anaerobic power and maximum strength [10]. Furthermore, resting energy expenditure, as well as perceptions of readiness to perform and cognitive function were also increased after MIPS [12]. In addition, the absolute energy expenditure may precede improvements in performance, since the higher ATP resynthesis rate is partly associated with increased enzyme activity, uptake of substrates/oxygen and/or removal of metabolites [13]. However, there is scarce evidence about the potential impact of MIPS on energy systems contribution and overall oxygen consumption during HIIE protocols, which are typically used by various populations to enhance fitness and body composition. Knowing which system is affected the most by MIPS ingestion could refine the prescription of this ergogenic aid to optimize specific training sessions and/or increase energy expenditure for particular populations. Therefore, there is a wide scope of research to be done in this area to promote both health and performance outcomes.

Our group recently investigated the physiological responses to HIIE (15 s of effort at the speed associated with 120% V̇O_2max_ interspersed with 15 s of passive recovery) after supplementation of a single ergogenic aid (capsaicin) that stimulates the sympathetic nervous system, lipid oxidation, and muscle contraction [14]. Results indicated no difference in the time spent above 90% of V̇O_2max_ (T90% V̇O_2max_) and energy systems contribution, although capsaicin significantly increased the number of efforts performed before volitional exhaustion of the participants. One may argue that using multiple ingredients could not only increase the number of efforts, and thereby the overall training load, but also raise energy expenditure and V̇O_2_ during HIIE [14]. Therefore, the aim of the present study was to investigate the acute effects of MIPS on the oxidative, glycolytic and adenosine triphosphate – creatine phosphate (ATP-CP) energy systems contribution, T90% V̇O_2max_ and excess post-exercise oxygen consumption (EPOC) magnitude in a HIIE session. We hypothesized that the MIPS would increase T90% V̇O_2max_ during HIIE and the EPOC after the session, conducive to an overall increase in energy expenditure.

## Methods

### Study design

This study used a randomized, double-blind, crossover design with experimental trials conducted in the morning (7 to 10 AM) and each visit separated by 72 h. During the first visit, anthropometric measurements and the incremental running test was performed to determine V̇O_2max_ and MAS. The following two visits, participants randomly consumed the MIPS or Placebo capsules one hour before performing a HIIE session. The V̇O_2_ and blood lactate concentration were monitored during the HIIE sessions to determine metabolic effort.

### Subjects

**Fig. 1:**
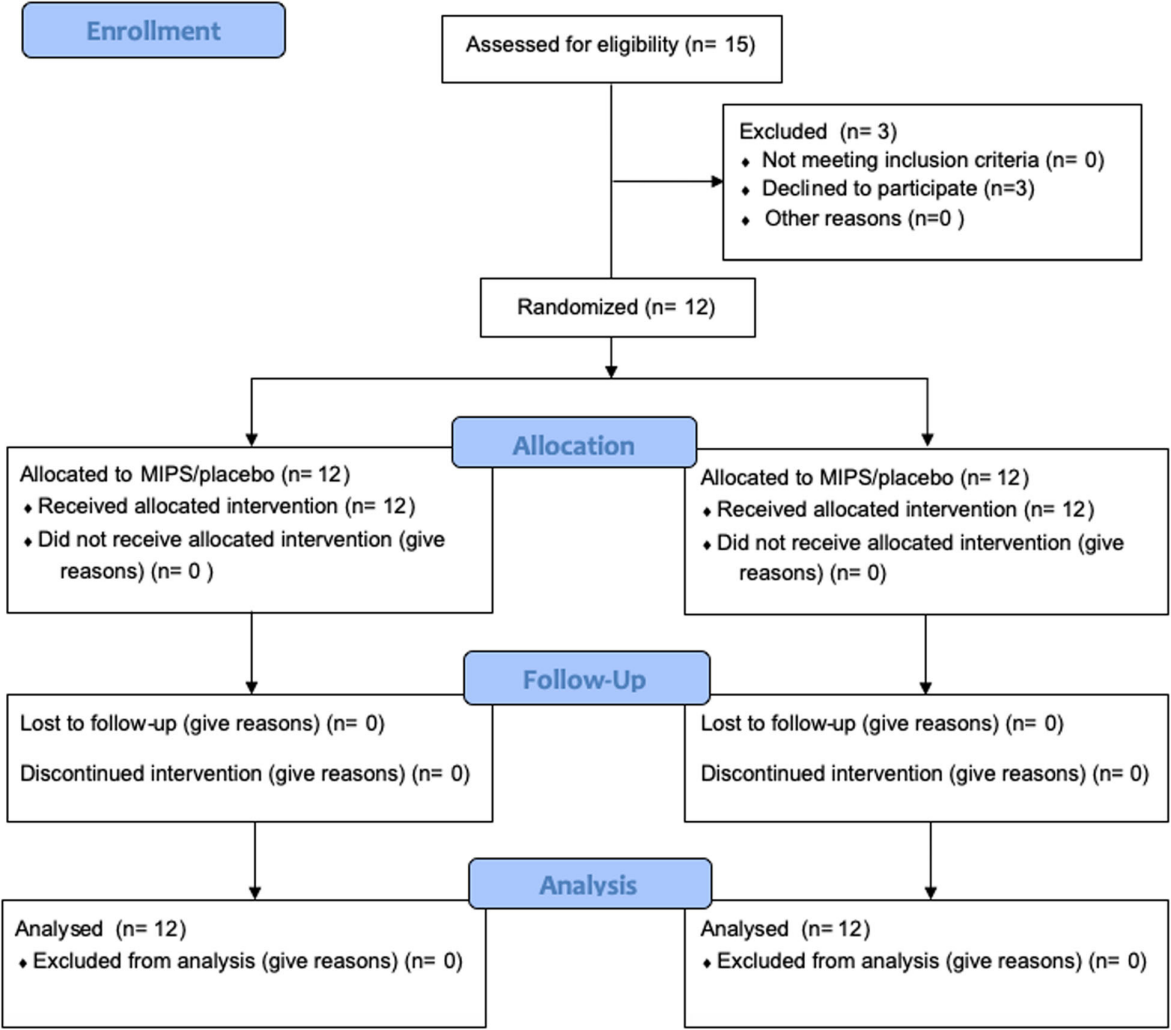
CONSORT diagram

Twelve physically-active and healthy men were recruited for this study. The interview considered the following criteria of inclusion: being male, between 18 to 35 years age and practice aerobic exercise for at least 6 months. The participants were also excluded if they presented any medical contraindications that might interfere with their performance on the exercise protocol. All participants signed a consent form and were informed about the purpose of the study and the possible risks involved. This study was conducted after approval by the Ethics Research Group of the Federal University of Piauí, Teresina-PI, Brazil (Protocol number: 3.169.545) and according to the 2008 Revision of the Declaration of Helsinki [15]. The data collection process (CONSORT diagram) is shown in Fig. 1.

### Dietary intake assessment, supplementation protocol and anthropometric measurement

Participants were instructed to consume the same breakfast and to record food and fluid intake 24 h before each HIIE session. Participants were also instructed to avoid coffee, tea, alcoholic beverages, or any other stimulants, supplement or ergogenic substance during the study period, and were instructed to maintain their regular food intake. The consumption of kilocalories and macronutrients was calculated based on the Brazilian food composition table (TACO) to ensure that intake was similar in both experimental trials.

The MIPS used in this study was a commercially available product supplied by the manufactuer *(Supley Laboratório, Matão, São Paulo, Brazil)*. The ingredients are listed in Table 1. The Placebo mixture (maltodextrin + juice Clight® containing maltodextrin, dyes, acidulants and flavorings, energy value = 34 Kcal, carbohydrates = 8.5 g) was diluted in 250 ml of water. Both placebo and MIPS mixture were identical in color and flavor. The MIPS or Placebo were delivered to the volunteers by a member of the laboratory who did not belong to the investigating research team to ensure a double-blind design. Participants ingested the substance in the laboratory one hour before the start of the experimental HIIE session. Body weight was measured using an electronic scale (Filizola PL50, Filizola Ltda., Brazil). A fixed stadiometer was used to measure height with an accuracy of 0.1 cm.

**Table 1:** Supplement ingredients

Serving Size: 3 Scoops (20 g)		
Amount Per Serving	Amount	% Daily Value
Calories	71	4%
Total Carbohydrates	8 g	3%
Calcium	180 mg	18%
Folic acid	500 mcg	208%
Vitamin C	250 mg	556%
Niacin	35 mg	219%
Vitamin B1	2 mg	167%
Biotin	20 mcg	67%
Vitamin B6	50 mg	3.846%
Vitamin B2	2 mg	154%
Vitamin B12	9.94 mcg	414%
***Proprietary Blend***^***a***^	***10.200 mg***	
Caffeine	400 mg	*
Creatine monohydrate	3.000 mg	*
L-Arginine	2.000 mg	*
L-Leucine	2.000 mg	*
L-Valine	1.000 mg	*
L-Isoleucine	1.000 mg	*
L-Tyrosine	800 mg	*

^*^Daily Value (DV) not established

### Incremental running test

Participants performed a maximal incremental test on a treadmill (Inbramed MASTER CI, Inbrasport®, Porto Alegre, Brazil) in an environment with controlled temperature and humidity. Expired gases were collected breath-by-breath with a silicon mask connected to the gas analyzer (Quark PFT - Cosmed®, Rome, Italy). The maximal incremental test was used to determine MAS for the prescription of HIIE sessions and V̇O_2max_ for the characterization of the sample. Participants performed a warm-up of 5 min walking at 5 km/h before test. The initial test speed was set at 6 km/h and increased by 1 km/h every 2 min. Treadmill inclination was maintained a 1% and the test was terminated when the participant reached voluntary exhaustion. Verbal encouragements were provided to ensure that every volunteer ran to exhaustion. The MAS was assumed as the final velocity of the test. When the participants failed to complete a stage, the speed was selected according to the following formula: MAS = complete final stage velocity + [(time, in seconds, remaining in incomplete final stage / 120 s) * 1 km.h] [16]. The rate of perceived exertion (RPE) and heart rate (HR) were measured during the test using the 6–20 point Borg scale [17] and Polar S810i (Polar®, Finland), respectively.

### High-intensity intermittent exercise (HIIE) protocol

For each trial session, participants performed a warm-up at 50% of MAS for 5 min. The HIIE involved running bouts of 15 s on the treadmill at 120% of the MAS, interspersed with 15 s of passive recovery. The inclination of the treadmill was maintained at 1% and the HIIE session was terminated when the volunteers achieved voluntary exhaustion. The total of sprint intervals performed and time to exhaustion under each condition were recorded.

### Blood lactate

Twenty-five microliters of blood was collected from the volunteer’s right ear lobe at rest, immediately post, and 3, 5 and 7 min post-exercise. Lactate concentrations were obtained using the Yellow Spring 1.500 Sport lactate analyzer (Yellow Springs, USA). The delta lactate (highest value minus rest values - [∆La^−^]) was utilized to compare conditions.

### Oxygen uptake

V̇O_2_ was measured at rest, during the HIIE sessions and for 20 min after the end of the sessions. For data treatment, the mean V̇O_2_ during the HIIE session was analyzed for 15 s in every effort and every for pause. The T90% V̇O_2max_ was determined from the average obtained in the 5-s periods due to the rapid changes in V̇O_2_ over time between sprint and pause periods in order to obtain greater sensitivity, and compared with the relative V̇O_2max_ values. EPOC was obtained by subtraction of V̇O_2_ from rest of the mean V̇O_2_ of recovery [14].

### Energy expenditure

The energy expenditure during the MIPS and Placebo trials was estimated from the contribution of the oxidative, glycolytic, and ATP-PC energy systems [18, 19]. The contribution of the oxidative energy system was estimated by subtraction of the resting V̇O_2_ from the mean V̇O_2_ calculated over the complete HIIE. For glycolytic energy system contribution, the Δ [La^−^] was converted into oxygen equivalents assuming that the accumulation of 1 mmol/L of lactate is equivalent to 3 mLO_2_/kg body weight [19]. Finally, the sum of V̇O_2_-time average during the HIIE recovery periods (ΣEPOC) subtracted by resting V̇O_2_ was assumed for the contribution of ATP-CP [20–22]. In addition, the fast component of EPOC (i.e., estimated using V̇O_2_ kinetics as the product of V̇O_2_ amplitude and time constant using a bi-exponential fit) was calculated for the last effort utilizing the software Origin version 2019 (OriginLab Corporation, Microcal, Massachusetts, USA) and added to ΣEPOC. The oxygen equivalents were converted into energy equivalents considering 20.92 kJ for each 1 L of O_2_ used [23].

### Statistical analysis

A power analysis (Power 1-β = 0.87) was performed a priori in G*Power software and indicated that effects could be detected with twelve participants. Data were reported as mean and standard deviation (SD). Data normality was verified using the Shapiro–Wilk test. The number of efforts performed, time to exhaustion, time above 90% V̇O_2max_, EPOC, dietary intake and oxidative, glycolytic and ATP-CP system contributions during MIPS and Placebo were analyzed by a paired t-test. For the lactate, a two way analysis of variance [group (MIPS versus Placebo) × time point (pre versus post)] was conducted followed by the Tukey’s post hoc test. Statistical significance was set at *p* <  0.05. The data were analyzed using SPSS version 22.0 (SPSS Inc., Chicago, IL).

## Results

**Fig. 2:**
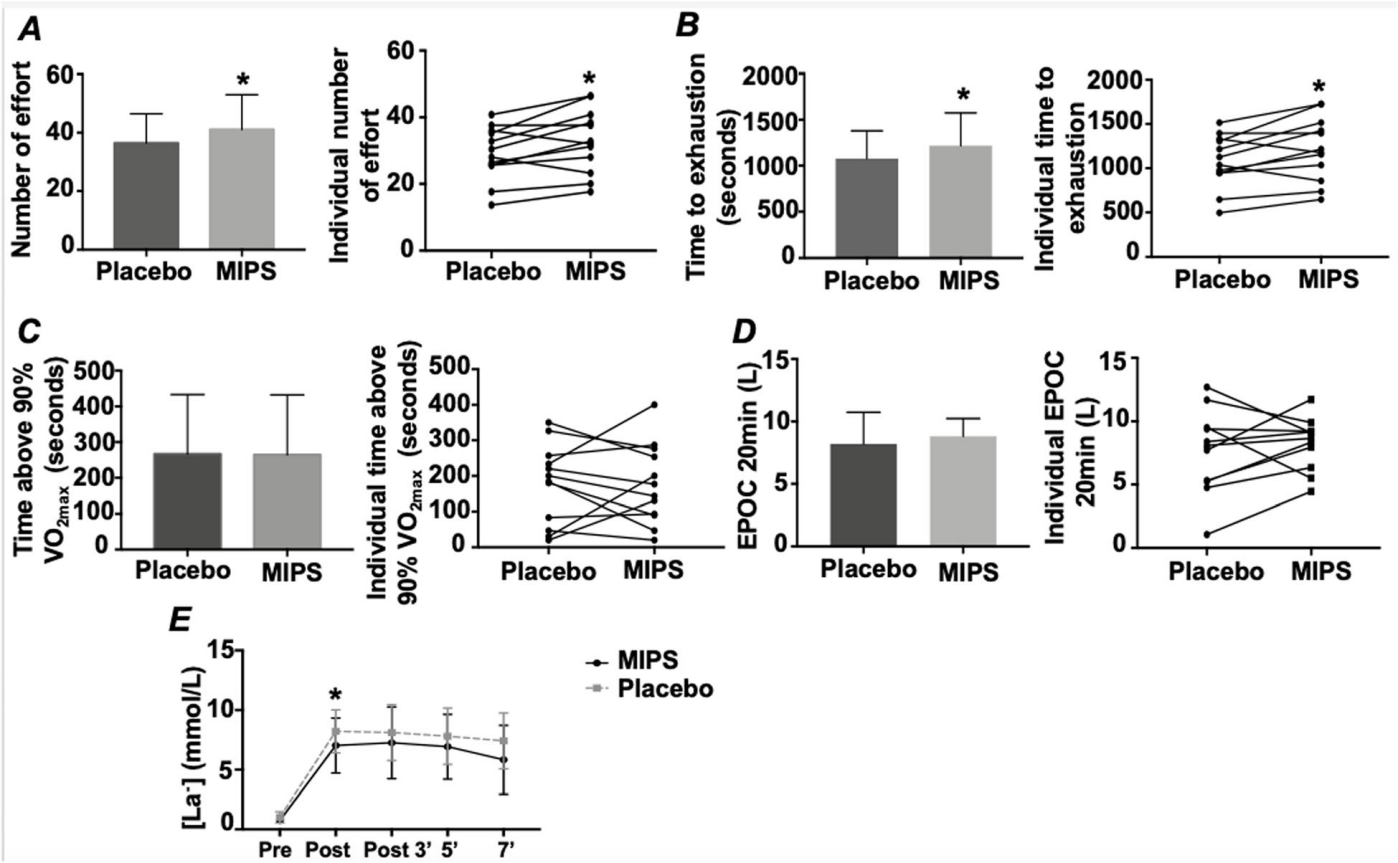
Performance responses in MIPS and Placebo condition. Number of efforts performed (**a**), time to exhaustion (seconds) (**b**), time spent above 90% of the V̇O_2max_ (seconds) (**c**), EPOC 20 min (L) (**d**) and lactate concentration (mmol/L) at pre, post, post 3, 5 and 7 min (**e**) in MIPS and Placebo condition. Data are presented as mean ± SD and individual response

Figure 2 shows the number of efforts performed, time to exhaustion, T90% V̇O_2max_ (seconds), EPOC 20 min and lactate concentrations of each experimental trial. Consuming MIPS significantly increased the number of efforts performed (MIPS: 41 ± 10 vs Placebo: 36 ± 12, *p* = 0.0220) and time to exhaustion (MIPS: 20.1 ± 6 min vs Placebo: 17 ± 5 min, *p* = 0.0226). There was no difference between MIPS and Placebo for both the T90% V̇O_2max_ (*p* = 0.9705) and EPOC 20 min (*p* = 0.4930). On the other hand, the V̇O_2max_ was lower in the MIPS condition when the number of efforts were equal between MIPS and placebo conditions. Although [La^−^] increased at all moments post-exercise compared with pre-exercise, there was no significant difference between supplements (*p* > 0.05).

Table 2 displays the dietary intake and macronutrient distribution 24 h before each trial. There was no difference in the dietary intake and macronutrient distribution of carbohydrate, protein and lipid (*p* > 0.05) of each experimental trial.

**Table 2:** Dietary intake and macronutrient distribution 24 h before each trial

Dietary intake	Placebo	MIPS	*p*
Carbohydrate (g)	281.7 ± 133.7	250.6 ± 139.9	0.148
Carbohydrate (g/kg)	3.7 ± 1.8	3.7 ± 1.9	0.151
Protein (g)	122.6 ± 36.6	115.3 ± 25.1	0.701
Protein (g/kg)	1.6 ± 0.4	1.5 ± 0.3	0.769
Lipid (g)	64.6 ± 30.4	66.4 ± 25.4	0.370
Lipid (g/kg)	0.8 ± 0.4	0.9 ± 0.4	0.455
Total intake (Kcal)	2.361 ± 895	2.127 ± 798	0.394
Total intake (Kcal/kg)	31 ± 12	31 ± 11	0.459

Data are presented as mean ± SD. * *p =* < 0.05 vs placebo condition

Table 3 displays the relative and absolute energy system contribution for each condition. Consuming MIPS significantly increased the absolute oxidative energy system contribution by 23.8% (*p* = 0.0163) and the absolute ATP-CP contribution by 28.4% (*p* = 0.0055) related to placebo condition. However, we only noted a tendency for a greater glycolytic system contribution after MIPS ingestion during the HIIE session (*p* = 0.0683).

**Table 3:** Energy system contribution for each condition

	Placebo		MIPS	
	Mean ± SD	Lower - Upper 95% CI	Mean ± SD	Lower - Upper 95% CI
*Relative*				
Oxidative (%)	67.4 ± 2.4	65.0–73.7	67.2 ± 1.2	64.5–69.1
Glycolytic (%)	1.1 ± 0.8	0.4–2.8	0.8 ± 0.7	0.5–2.6
Phosphagen (%)	31.7 ± 2.1	25.9–33.8	31.9 ± 1.0	30.1–33.6
*Absolute*				
Oxidative (Kcal)	457.4 ± 156.2	250.2–687.0	565.9 ± 168.7^*^	296.8–810.1
Glycolytic (Kcal)	7.4 ± 3.0	3.9–12.2	8.2 ± 2.5	5.2–11.9
Phosphagen (kcal)	210.7 ± 64.0	117.1–307.6	271.0 ± 80.1^*^	145.6–380.0

Data are presented as mean ± SD and lower-upper 95% CI in placebo and MIPS conditions. * *p* = < 0.05 vs placebo

## Discussion

To our best knowledge, this is the first study to investigate the acute effects of MIPS consumption on key physiological responses and performance during HIIE. The main findings of this study were that consuming MIPS one hour before exercise significantly increased time to exhaustion during 15-s running bouts at 120% MAS, and upregulated energy expenditure. However, our initial hypothesis that T90% V̇O_2max_, EPOC, and glycolytic contribution would increase during HIIE after MIPS consumption was refuted.

Minimal research exists on the effects of MIPS on time to exhaustion [9]. In the present study, healthy male participants were able to perform 5 more efforts after ingesting 250 mL of MIPS compared to an isocaloric placebo. Our results are in accordance with a previous study showing that MIPS (Carnosyn® Patented Beta Alanine, Choline Bitartrate, L-Tyrosine, L-Glycine, Taurine, L-Carnitine Base, Beet Root Extract (*Beta vulgaris*) (High in Nitrates), Hawthorn Berry Powder (*Crataegus pinnatifida*) (Fruit), Agmatine Sulfate, Caffeine Anhydrous, Huperzine A 1% (*Huperzia serrata*) significantly increased the total distance covered during a 25-s maximal running test on a treadmill (MIPS: 94.0 ± 6.7 vs placebo: 93.01 ± 7.5 m, *p* = 0.039) [24]. In addition, acute ingestion of MIPS (26 g of a powder containing an energy matrix (2.05 g of caffeine, taurine, glucuronolactone), a proprietary amino acid matrix (7.9 g of L-leucine, L-isoleucine, L-valine, L-arginine and L-glutamine), 5 g of di-creatine citrate, and 2.5 g of β-alanine) mixed with 500 mL of water during continuous 3 h post-absorptive state, also increased exhaustion time by 12.5% compared to a placebo during an continuous exercise at 70% of V̇O_2max_ on a treadmill [25]. Interestingly, the MIPS of all the studies presented above use caffeine as their “flagship” ingredient likely due to its potent and well-known acute ergogenic effects [26]. It has been robustly demonstrated that caffeine positively influences in central fatigue and muscular endurance by its direct effect on muscle anaerobic energy provision and its ability to increase muscle contractility [27, 28]. Caffeine acts as an adenosine receptor antagonist and has been shown to acutely improve cognition as well as performance during endurance, power, and resistance exercise when consumed in dosages between 3 and 6 mg/kg bodyweight [9, 29]. The caffeine content of this MIPS (400 mg) is in that acceptable range for most individuals. A recent systematic review and meta-analysis of forty-six studies about caffeine reported a small but clear effect on endurance performance when taken in moderate doses (3–6 mg/kg) as well as an overall improvement in mean power output (3.03 ± 3.07%; effect size = 0.23 ± 0.15) and time-trial completion time (2.22 ± 2.59%; effect size = 0.41 ± 0.2) [28]. Therefore, the caffeine contained in the MIPS tested in the present study may likely have contributed to the enhancement in performance at high intensity.

Tyrosine is a nonessential amino acid which plays an essential role in production of catecholamine neurotransmitters, including epinephrine, dopamine and norepinephrine. Coull et al., (2015) reported that tyrosine supplementation improves cognitive function during a simulated intermittent soccer performance test (iSPT), with no change in performance response [30]. Tyrosine supplementation alone seems to have little influence on performance, however, its cognitive action associated with other ergogenic resources deserves attention. The B-vitamins (thiamin, riboflavin, niacin, vitamin B6 and vitamin B12) are necessary in the energy-producing pathways of the body, while folate and vitamin B12 are required for the synthesis of new cells, such as the red blood cells, and for the repair of damaged cells [31]. Therefore, individuals with a vitamin deficit in the diet may be favored with consumption of MIPS, even though this is not the main objective of supplementation. On the other hand, athletes who have poor diets, those restricting energy intakes or eliminating food groups from the diet, should consider L-arginine supplementing. L-arginine is an amino acid that is a precursor required for the synthesis of nitric oxide [32]. L-arginine would therefore favor muscle blood perfusion and increase of nutrient availability to muscle, and favor greater release of metabolites, such as lactate and ammnonia, which are related to the muscle fatigue [33]. However, almost all studies in trained athletes reported limited efficacy in improving blood flow or exercise performance with acute and chronic arginine oral supplementation [33]. Another important compound associated to performance is the creatine monohydrate. Although the acute effect of creatine supplementation be unknown, the supplementation of creatine monohydrate over time is able to increase performance in high intensity exercises, prevent injuries and help in the recovery process [34]. In addition, the effects of creatine monohydrate have been studied as a possible therapeutic strategy in neurodegenerative, metabolic and behavioral diseases [34]. Thus, although MIPS has these ingredients, they probably played a marginal role in the results found due to acute supplementation and caffeine is probably the only ingredient that may have contributed.

The energy contribution of HIIE protocols varies according to ATP demand and oxygen availability. The contribution of the aerobic system in the present study was greater than that of the anaerobic system (67% vs 33% for MIPS condition). These relative results are in agreement with studies using the same [14] and a similar [22] protocol. More importantly, the MIPS in the present study was effective at increasing the oxidative and ATP-CP contribution, and there was also a tendency for an increased glycolytic contribution (*p* = 0.068). The study conducted by de Freitas et al. (2019) also used a protocol involving 15-s repetitions at 120% MAS, with capsaicin supplementation, a natural bioactive substance that stimulates the sympathetic nervous system, lipid oxidation, and muscle contraction. This study did not observe any change in energy system expenditure, EPOC and T90% V̇O_2max_, despite a longer time of exhaustion with capsaicin (+ 14.6% efforts). It is interesting to note that, although using different participants with probably different fitness levels, the number of efforts performed after ingesting a single dose of capsaicin was higher than after consuming MIPS (+ 13.8% efforts) as done in the present study. However, the higher performance with capsaicin did not reflect a higher metabolic demand [14]. Thus, although the present study did not show significant changes in EPOC and T90% MAS after MIPS ingestion, our initial hypothesis that MIPS could be a relevant strategy to increase energy expenditure is confirmed based on the findings of greater contribution of the oxidative system. These results are therefore of interest to people who wish to increase energy expenditure in HIIE sessions. The chronic effects of MIPS ingestion on body fat and cardiorespiratory fitness will have to be ascertained, especially in overweight populations. Furthermore, applied sport science studies will be required to explore the impact of MIPS in different sports and activity modalities.

The main limitation of the study was the absence of comparison between the MIPS and caffeine alone. In fact, although MIPS appears ergogenic compared to a placebo, a caffeine-equivalent control group would have ruled-out the impact of this substance alone vs. others and strengthened our understanding of the efficacy of MIPS. Nonetheless, based on these data, there is a need to explore the potency of combining MIPS with high-intensity interval training to assess the chronic effect of this type of supplementation.

## Conclusion

Acute ingestion of a multi-ingredient supplement containing vitamins, amino acids and caffeine is a relevant strategy to increase energy expenditure and time to exhaustion during HIIE.

## Data Availability

Data and publication materials can be provided upon request. Please contact corresponding author for this information.

## Abbreviations

ATP: Adenosine triphosphate
ATP-CP: Adenosine triphosphate – creatine phosphate
CAPES: Coordenação de Aperfeiçoamento de Pessoal de Nível Superior
EPOC: Excess post-exercise oxygen consumption
HIIE: High-intensity intermittent exercise
HR: Heart rate
La^−^: Lactate
MAS: Maximal aerobic speed
MIPS: Multi-ingredient pre-workout supplementation
RPE: Rate of perceived exertion
SD: Standard deviation
TACO: Brazilian food composition table
V̇O_2max_: Maximal oxygen uptake
